# Vulnerability and Entitlements

**DOI:** 10.1007/s11196-022-09942-1

**Published:** 2022-11-01

**Authors:** Bryan S. Turner

**Affiliations:** Australian Catholic University, North Sydney, NSW 2060, Australia

**Keywords:** Dignity, Human rights, Citizenship, Vulnerability

## Abstract

The article examines the merits of both human rights and citizenship as systems to protect vulnerable individuals. The idea of vulnerability is presented as a more reliable concept than the dignity of the individual in comparative research. The body is basic to vulnerability.

## Introduction: The Argument

This article compares human rights and the social rights of citizens. I consider the normative basis for universal human rights claims, namely the dignity of the human person. I argue that ‘the dignity of the person’ cannot be neatly translated between cultures and raises familiar problems about cultural uniqueness. By contrast, humans share a common vulnerability, relating to their embodiment, their inevitable ageing, and ultimate demise. Vulnerability therefore can serve as the normative basis for rights claims. In conclusion, the limitations of both systems of rights are considered.

The connections between vulnerability and rights have been a topic of some interest in the general literature on rights. For example, Keohane and Nye [22] in *Power and Interdependence* in their discussion of the costs of interdependency introduced two dimensions–sensitivity and vulnerability in which the latter refers to whether a society has the ability to enforce the policies that would be necessary to reduce or eliminate the costs arising from such threats. In response to their approach, Peadar Kirby [23] in *Vulnerability and Violence* argues that their concept of vulnerability is far too limited to capture the complex and diverse nature of vulnerability. He adopts the idea of a ‘risk society’ [4] to examine the various ways in which our insecurity is magnified by risks related to technological change and globalization. He understands our vulnerability to be evident in variety of contexts: economic, financial, political, social, environmental, and personal. These studies of rights and risks have made important contributions to the study of growing human insecurity and vulnerability in modern societies. In my view, there is however a strange absence to their approach, namely that their subjects are strangely disembodied. To understand our existential threats, we need to attend to the peculiar physical and mental vulnerability of humans. Rape, torture, and starvation, that typically attend human rights abuses, occur through the medium of our bodies [52]. The definitive text on this issue especially in relation to torture is Elaine Scarry [33] *The Body in Pain.*

The idea of human rights requires an underlying and universal basis that is credible and guarantees entitlements to claim the safeguards that human rights offer to individuals who are in need of some form of protection. To begin, how do we confirm the validity of the idea of ‘the dignity of the human being’? The question of sovereignty comes into play with both the protection and enforcement of human rights and citizenship. Effective citizenship requires a democratic sovereign state that is responsive to the needs of citizens provided the franchise and the electoral machinery have the capacity to dismiss errant governments. Although human rights are understood to be universalistic claims, in practice they typically depend on a sovereign state to uphold rights claims. As a result, the borderlands between sovereign states inevitable become sites of contestation often resulting in violence [24]. There are some locations where neither human nor citizenship rights have any purchase, and the law is suspended in what Georgio Agamben [1] calls the ‘Exception’. His most powerful example of what he calls ‘bare life’ describes the detention camp at Guantanamo Bay.

Social citizenship and human rights, as legal and political institutions, are the main candidates as systems of rights that protect individuals and offer some modicum of security. Both systems have severe limitations which I explore in this article. For example, without membership within a sovereign nation state, many individuals, such as refugees, are highly vulnerable. Human rights may have more relevance to a global world, but they are difficult to fund and to enforce. These limitations are well known, but the limitations of *both* systems–human rights and citizenship—are typically considered separately. In short, while I may have achieved some clarification of the problems, there is no neat or definitive set of solutions to the issues that are discussed in this article.

## The Historical Background to Rights

Whereas human rights are essentially a product of the twentieth century, the idea of citizenship has a considerable ancestry. Aristotle defined it at the beginning of his *Politics* Book III i.2. In a passage that sounds strikingly modern, he said that citizens are not such just because they are born in a place and by contrast alien residents are not true citizens. The special characteristic of a citizen is that ‘He who has the power to take part in the deliberative or judicial administration of any state is sad by us to be a citizen of that state; and a state is a body of citizens sufficing for the purpose of life’[3:102]. I use ‘he’ in this quotation from Aristotle, since women were not Greek citizens. In our contemporary terms, citizenship would include jury service, the right to vote, service in the military, and holding a public office. Of course, Aristotle knew nothing about human rights. He was describing the rights and duties of men, who as warriors were periodically called upon to defend or to expand the *polis.*

The modern idea of citizenship comes from the American Revolution (1775–83) and the French Revolution (1789–1799) and becomes established with the growth of the nation state and the basic principles of national sovereignty. Whereas the American Revolution separated church and state guaranteeing individual freedoms, the French Revolution enforced a policy of dechristianization that overrode religious freedoms. The secular notion of citizenship continues into modern times via the American Declaration of Independence, the Constitution, and the Bill of Rights, which refer to the ‘protection of divine Providence’. The Constitution, substituting ‘happiness’ for John Locke’s ‘property’, famously promoted the ideas of ‘life, liberty, and happiness’. A central theme therefore of the Revolution was the liberty of the citizenry. Benjamin Constant (1767–1830) played an important role in France and America in formulating the basic ideas of the liberal values of the citizen, but towards the end of his life he became disappointed with the emphasis on individual liberties at the expense of social duties and responsibilities [31]. The notion came under criticism from Constant who concluded it encouraged selfishness and dissolved communal connections. In my terms, citizenship involves rights and duties if civility is to flourish.

The idea of human rights has been around, at least in modern times, since the National Constituent Assembly of France issued ‘The Declaration of the Rights of Man and the Citizen’. The Assembly declared that ‘the natural and imprescriptible rights of man’ were essentially ‘liberty, property, security, and resistance of oppression’. These rights however in turn presupposed the existence of sovereign states that could defend and guarantee them. Furthermore, the nation was understood to be the ‘source of all sovereignty’.

The French Revolution was the occasion for major developments in political and legal theory. For example, Edmund Burke in *Reflections on the Revolution in France* in 1790 was clear about the differences between the rights of man and the citizen in the French Revolution. For Burke, the Rights of Man were abstract, vague, and flimsy by comparison with the empirical and established rights of an Englishman whose rights were based on tradition and custom. Institutions cannot be invented or legislated; they grow and evolve, responding to natural rather than artificial circumstances. Against revolutions of any sort, he argued ‘it is with infinite caution that any man ought to venture upon pulling down an edifice which has answered in any tolerable degree for ages the common purposes of society or building it up again without having models and patterns of approved utility before his eyes’ [7]:70]. Burke treated ‘natural rights’ as sacred and hence not to be radically refashioned by revolutionary intervention:

‘The rights of men natural rights of mankind are indeed sacred things; and if any public measure is proved mischievously to affect them, the objection ought to be fatal to that measure…. These things secured by these instruments may, without any deceitful ambiguity, be very fitly called the chartered rights of men’ [8].

Burke did not reject the role of a landed aristocracy in creating a stable society, but he condemned the failure of political leadership that lay behind the American war of independence. It was better he argued to grant America independence outright than to fight over it. In 1777 in *A Letter to the Sheriffs of Bristol on the Affairs of America* (11,36), he wrote ‘I should expect ten times more benefit to this kingdom from the affection of America, though under a separate establishment, than from her perfect submission to the crown and parliament, accompanied with her terror, disgust, and abhorrence. Bodies tied together by so unnatural bond of union as mutual hatred, are only connected to their ruin’. In these exchanges, he formulated an early version of the idea thaT the common law better expresses the lived historical experience of a community of individuals than any abstract system of laws.

Why dwell on Burke – a writer much (wrongly) criticised for his conservatism? There are at least two reasons for introducing this discussion of Burke. Firstly, Burke [7] in *Reflections on the Revolution in France* in 1790 offers an important defence of customary entitlements in a common law tradition with the backing of a representative assembly – albeit it imperfect and elementary. Burke was a conservative, but nevertheless willing to accept the need for change for example in the American colonies and in the management of Indian affairs. In fact, he was the first to offer a political philosophy of conservatism. Burke also enters modern debates about rights in so far as his ideas influenced Hannah Arendt – one of the most important twentieth century political theorist of rights. Controversially, Hannah Arendt in 1951 in *The Origins of Totalitarianism* appears to have agreed with Burke. She recognized that once the ‘transcendent measurements of religion or the law of nature have lost their authority’, Hitler’s slogan (‘Right is what is good for the German people’) is inescapable as a description of how the law operates. Consequently, these observations are ‘an ironical, bitter and belated confirmation’ of Edmund Burke’s political philosophy [2]: 299). In her response to the plight of Jews in Nazi German, she argued that, without citizenship and membership of a sovereign nation state, there was no authority able to protect human beings. Human rights without a sovereign state were merely abstract claims.

Burke remains important therefore in that he presents a defence of sovereign states as the effective basis of rights claims. While in this article I emphasize membership of a state as a defence of rights, other authors have noted that with the growth of globalization, human rights are more in tune with globality than citizenship [54]:129]. However, given the contemporary crises in the global system – authoritarianism, pandemics, terrorism, and warfare – the global world perhaps looks less attractive. It also underlines the vulnerability of humans in the current collection of catastrophes involving pandemics, wars, famines, and civil unrest.

The basic argument here is that citizenship and human rights are distinctively different rights regimes, and they are not easily reconciled. In fact, the two systems are currently very much at odds with each other over the issue of national sovereignty. This may not be an original idea [46]. Hans Joas, who has contributed to the sociology of war and the universalism of human rights regimes asserts in *War and Modernity* [21:23]’ the central conflict of values in this sphere today is the conflict between national sovereignty and the universalistic claims of human rights.

Citizenship is an exclusive and narrow form of membership of a political system, typically a sovereign state, based on reciprocal rights and duties. Its origins are secular and bound up with the emergence of state systems and national sovereignty. Citizenship is rarely comfortable with cosmopolitanism and in recent years is increasing associated with ethnic nationalism. Human rights can be and are increasingly legislated into national legal frameworks, and human rights laws and values can be employed to criticize how citizenship is failing in certain circumstances. It is generally recognised that sovereignty presents serious problems for the recognition and enforcement of human rights. Those individuals, who are not bona fide citizens of a sovereign state, have no legal entitlement to citizenship rights, but it is also difficult for them to access human rights and have them enforced. Genocide is probably the extreme example where the defence of sovereignty stands in the way of international bodies enforcing human rights [35]. Human rights can be regarded as a system of last resort in typically providing some protection for persons who are the victims of war and genocide. Human rights have failed to enforce justice where states claim immunity from external legal oversight as in the case of the Uyghurs in modern day China or the long-standing marginalization of and discrimination against gypsies in Europe.

Human rights are also unlike citizenship right in that there is no correlativity between rights and duties. There is no declaration of human duties. Attempts have been made to develop such a declaration of human duties, but that has not gone very far. The (so-called) Valencia Declaration of Human Duties and Responsibilities (1998) was in part a celebration of the 1948 Declaration of Human Rights and an attempt to underpin the idea of universal rights with the recognition of human duties. The duties of citizens have historically included an obligation to vote in elections, to undertake military service in wartime, to serve in juries, ad above all to pay taxes.

In the development of the idea of personal dignity, the role of the Roman Catholic Church is seen as important [30]. On Christmas Day 1942 Pope Pius XII issued five peace points. The first was an appeal to the ‘dignity of the Human Person’ which was based on a Christian understanding of ‘personalism’. In fact, the idea of ‘the person’ has been seen as specifically a construction within Christianity. Marcel Mauss argued that the notions of the ‘self’ and the ‘person’ have a distinctly western history starting with Roman law in which *personne, res,* and *actiones* were foundations. In Roman law, the person emerged as a being entitled to rights. It was much later that the idea of individual consciousness began to emerge with the evolution of Christianity. Mauss concluded that the modern notion of the ‘human person’ is ‘still the basically Christian one’ [28]:19]. Much of the subsequent philosophical work on the idea of the person was undertaken by Jaques Maritain who appealed to the natural law tradition to avoid the individualistic and secular notion of the person [27]. Moyn defends the influence of the Christian notion of the person but argues that the Church ultimately failed to promote and defend a universal system of human rights. It was not until after Vatican II that the Church began to accept the idea of ‘religious freedom’ as applying to all forms of religion. Moyn argues that the acceptance of human rights was delayed by the political failure to recognise the true extent of the Holocaust. Furthermore, the movement for post-colonial national independence after WWII did not leave much opportunity for promoting human rights internationally as new postcolonial governments focused on the considerable domestic problems they faced. [29]:69] concludes ‘Postponed in the focus on declaring rights, the prospect of moving to legally enforce human rights across borders that a few observers still considered a live possibility as late as 1949 was dead by 1950’.

## Vulnerability: Person and Body

Most of these approaches to human rights ignore the embodiment of human beings as fundamental to their existence and wellbeing. In previous publications I have argued that rights talk in general terms emerged in response to recognition of our inescapable vulnerability. Obviously, we can suffer from mental vulnerability when confronted by real or imagined suffering. In this discussion however, I focus exclusively on bodily vulnerability. The word ‘vulnerable’ and the condition of ‘vulnerability’ come from the Latin word *vulnus* for a ‘wound’. In these terms, we are fundamentally wounded creatures. The obvious objection is that some people are more vulnerable than others. However, death is the fate of all of us, and hence death and dying are fundamental to vulnerability and the shared root of our existential uncertainty.

There are obvious limitations to the ideas of ‘dignity’ and ‘person’. The most problematic is the cultural specificity of both terms. We need to take seriously the historical background and etymological provenance of words. It is interesting, and perhaps ironic, that ‘person’ is from *persona* or the mask worn by an actor in a play to disguise their actual identity. It also suggested disguise or semblance. ‘Person’ thus disguised the identity of the individual behind the mask. The origins of ‘dignity’ connect the quality of dignity to a dignified person that is somebody with status or rank, who thereby also enjoy gravity. In its original meaning, people without rank lacked dignity. In a modern society, the dignity of the ‘person’ has been disconnected from the idea of the prestige or honour of a station in society. Apart from its cultural ambience, there are additional issues. Life begins with fertilization, but at what stage does a human foetus acquire personhood – at the point of birth or with maturity? Contemporary campaigns against abortion, based on interpretations of the creation story in the Old Testament, claim a person exists before their delivery into the world. Campaigns in support of a woman’s right to abortion typically reject religious interpretations of personhood and defend a woman’s right to control over her own body. As with all animal life, a human foetus is an unborn offspring of human parents, and it is clearly vulnerable but lacks personhood and dignity. To remain consistent, my argument requires that the foetus is recognized as obviously vulnerable. Many at birth are wounded and may be disabled for life. However, the mother for various reasons such as poor health or being the victim of rape is also vulnerable and may decide to assert her right to control her own body against the unborn foetus.

Ageing is also an important feature of our embodied vulnerability. There is ample modern literature about positive ageing, but such optimistic notions often overlook the trials and tribulations of ageing–immobility, incontinence, loss of memory, joint pain, impairment of vision and so forth. In a recent publication in *The Evening of Life* [51] the American actress Betty Davis was quoted as saying–‘old age ain’t no place for sissies. Consequently, I am critical of the claims of ‘regenerative medicine’ which is expensive and beyond the reach of the majority of citizens [11]. For the overwhelming majority, bodily malfunctions accumulate over time and to some extent with modern scientific medicine we can cope with some of these difficulties, but we cannot expect to live forever [46]. Most people as they age must struggle over many years to cope with this vulnerability. Sociologists have to take our embodiment far more seriously than has been characteristic of sociological theory in the past [43]. In fact, sociologists might be said to be averse to the idea of ‘nature’ and, because of its long running battle with Darwinism, contemporary sociology has downplayed biology as a relevant science and evolution as a basis for a theory of social change. By attending to issues concerning our embodiment, the notion of vulnerability as the fundamental condition of human existence appears to be inescapable. The fact of our vulnerability erupts across all human activity. We are wounded by sarcasm and unkindness, but equally by falling off a ladde [41].

Arnold Gehlen (1904–1976) was an important influence in the growth of philosophical anthropology [46], which provides a valuable framework for any theory of human vulnerability. For [14],^15^], human beings are characterized by their instinctual impoverishment which means that humans depend on building institutions rather than instincts to allow them to cope with challenging environments. Humans are described as ‘deficient beings’ (*Mängelwesen*). Human beings are characterized by their instinctual impoverishment which means that humans depend on creating institutions to allow them to survive in diverse and challenging environments. They lack the instincts that could make their lives satisfactory and comfortable in a given environment. Humans are therefore characterised by their ‘world openness’ in the sense that they can survive in a wide range of natural environments from the American plains to the inhospitable forests of Siberia. This environmental openness is a distinctive advantage over other animals whose survival is tied to specific environments. However, institutions are fragile, requiring constant correction, management, and reinvention. These social institutions allow for a ‘stabilised relief of affections’, whereby in terms of important aspects of human existence such as sexuality, fear can be managed through becoming institutionalised. The building of institutions, including rites and rituals, was important in early human development where communal rituals were valuable in hunting success and early forms of agriculture. Such rituals also imposed the norms that regulated sexuality, marriage, death, and burial. With ‘the death of God’ in the West as announced in 1908 by Friedrich Nietzsche [31] in *Ecce Homo*, the institutionalization of society becomes even more critical. For Gehlen, modern societies are characterized by a ‘subjectification’, whereby the institutional controls are weak and social order becomes precarious. Gehlen is also credited with inventing the idea of *post-histoire* to capture the idea that everything is in transition, and nothing stays the same – hence our lives become more uncertain and precarious. His argument that in modern societies individuals suffer from ‘overstimulation’ and the negative effects of ‘de-institutionalization’ became popular slogans on the Left in Germany in the 1960s. In traditional societies, the gods also imposed the rules that regulated sexuality, marriage, death, and burial.

Although the rich and powerful might be assumed to enjoy considerable resources to mitigate their vulnerability and exposure to danger, our embodiment is an important condition of a shared equality. We are all exposed to the ageing process and attendant trials and tribulations of existence. But let us take some more routine examples. Given our upright posture, backache and back injury are common experiences. Our skin is easily penetrated, and we have no warm coat of fur against the winter cold. We suffer the usual annual round of influenza and pneumonia if we are already frail. Another example is the routine experience of toothache. I quote from Shakespeare’s *Much Ado about Nothing* [55] ct 5, Scene 1,31–38:

*Leonato*: I pray thee peace. I will be flesh and blood; For there was never yet philosopher, that could endure the toothache patiently.

In response to the argument regarding toothache, critics have argued the incidence and severity of toothache is variable [53]: 70–71]. In other words, toothache and other aches and pains are variable between individuals and consequently do not support a claim regarding a shared vulnerability. Against my broad notion of vulnerability, it can be argued that our mortality cannot be considered as just one more item on a list of conditions, but rather death must have a unique status in relation to other components of our vulnerability. Although these examples of backache and toothache may be less than completely compelling, I take our inevitable ageing and death as indisputable evidence of our inescapable and shared vulnerability.

This argument about the inevitability of ageing and death has however been challenged by biomedical gerontologists such as Aubrey de Grey, who was a founder and briefly the Chief Science Officer of SENS, which is a research foundation committed to the development of anti-aging research. Over the last 20 years, SENS has received significant investment for research. He is the co-author of *Ending Aging* [11] in which they argue that our ageing is an effect of a limited number of conditions for which there are various solutions- medical and technological. It is as if our life extension is basically an engineering problem. His defence of medical intervention to reverse ageing is convincing in a mundane fashion, when for example at conferences he asks the audience ‘Anybody here wants to die early from cancer, diabetes, or stroke?’ Few people offer to put up a hand. His scientific ambition is to demonstrate that, within this century, people in their mid-life will not or need not at least, die from such conditions that are currently our common lot. In all probability, humans will die from such mundane accidents as falling off a ladder in the prime of life. It is true of course with modern medicine, better sanitation, and social improvements such as housing and sewerage that human life has been extended. The third wave expansion of democracies in the last century that have been described by Samuel Huntington [19] contributed to global improvements in health that were the foundation of expanded life expectancy. However, major improvements in life circumstances in the democracies have not radically changed the final stages of the ‘existential ladder’ [47]. While medical and social improvements have ameliorated suffering and increased life expectancy, there is at present no convincing evidence that we can even contemplate living forever [46].

Human ageing is the outcome of a long process of evolutionary development and de Grey’s critics argue that the idea that these processes can be easily reversed is both naive and arrogant [17]. Bioethicists such as Ezekiel Emanuel have claimed that, given the ongoing devastation of the environment and limitations on the extent of recycling waste, we cannot avoid rationing resources and that a life of 75 years can be regarded as ‘having lived the good life’. He has argued that, with the steep decline in the quality of life after 75 years, the quest for unlimited life cannot be justified on ethical or social grounds. COVID-19 raises many questions about whether a society should invest resources in young generations embarking on their first stages of life or in frail citizens moving inexorably towards the end of their lives. These questions can only be answered successfully, or at least seriously considered, in the context of the limitations on available resources.

## Citizenship versus Human Rights

In this section of my discussion, I treat citizenship and human rights as separate systems of entitlement. In the modern world, citizens may of course have the benefit of both regimes of rights. Both the rights of citizens and the rights of human beings have arisen and evolved to give us some relief from the trials and constraints that attend our vulnerability. While the political rights and legal entitlements of citizenship have evolved over past centuries, the specifically socio-economic components of citizenship are relatively modern and are connected to the growth of industrial capitalism and the emergence of the modern working class. Welfare rights have been fundamental to the expansion of social citizenship. Early forms of social security and welfare were developed by Otto von Bismarck (1815–1898) in Germany with his Accident and Health Insurance Bills that eventually became law in 1883–4. These were followed in 1889 with a measure to cover old-age and disability insurance cover (Gall 1986). This legislation was designed to limit the appeal of socialism and to win the working class to his side [41]. [42] in his famous history of Bismarck wrote ‘German social insurance was the first in the world and has served as a model for every other civilized country’. The development of modern welfare citizenship is a product of state responses to the social and economic consequences of World War II. In the United Kingdom, welfare rights evolved during World War II and were expanded after the War under the influence of Keynesian economics. Two developments were significant namely The Education Act was steered through Parliament by R.A.Butler in 1944. It created free secondary school education to the age of 15, thereby establishing a universal education system. The legislation to establish the National Health Service was even more far reaching in its long-term impact. These developments were analysed and celebrated by Ralf Dahrendorf and T.H.Marshall [47, 48].

If Britain is often regarded as the cradle of the welfare state following the Beveridge Report (1944) and Keynesian economic policies, then the United States is often regarded by contrast as the cradle of individualism with little development of any collective provision of welfare. The contrast is perhaps well illustrated by Judith Shklar’s influential *American Citizenship* [36] in which she argued that most perspectives on citizenship in political philosophy had overlooked the importance of employment and earning in the foundation of ideas about citizenship in colonial America. The founding fathers feared the twin issues of both slavery and aristocracy. Slavery involved a loss of human status and dignity, while the aristocrat was associated with luxury and idleness. Thus ‘We are citizens only if we “earn”’ [36]: 67].

However, both of human rights and citizenship have well-known difficulties. Citizenship is not suited to changes brought about by globalization, because, being wedded to unitary sovereignty, it has struggled to come to terms with globalization, migration, multiculturalism, and social diversity. The judicial establishment of rights is no guarantee of their implementation or survival. The impact of Reagonomics and Thatcherism in the 1970s was to install a period of neoliberal policies that introduced various policies to privatize many public services, to limit the role of trade unions in the economy, and in cultural terms to promote individualism. One result was the financialization of capitalism. In this transformation of capitalism, fortunes are made through speculation rather than by industrial production. Industrial profits are not reinvested in capital, technology, and wages. This form of capitalism can be said to promote profiting without producing. New profits are associated with the return on dividends, interest on international bank loans, and retirement and investment funds [26]. The process resulted in a new culture which for some produced a more creative social environment and for its critics it unleashed anti-social greed. While some economists who favour liberalization have argued that it produces benefits, others have argued that it is detrimental to economic productivity, that the financial sector is too large and that there is a problematic divide between the ‘real economy’ and the ‘artificial economy’ in which finance can contaminate the working of the economy.

These changes in capitalism lay behind the global crisis of 2008–2011. The collapse of Lehman Brothers in 2008, following the failure of two hedge funds in Bear Stearns in 2007, caused a profound shock to the American economy that had global consequences. One result was the adoption of ‘austerity packages’ the European Union with draconian constraints imposed on weak economies such as Greece and Spain. One result was the rise of new left-wing and populist political movements in Spain and Greece [39]. The result of this development from the 1970s to the early decades of this century was to erode the sense of collective responsibility and citizenship rights. I refer to this erosion of social rights as resulting in ‘weak citizenship’ in which citizens begin to resemble denizens whose connection to the national community is weak and declining [48].

What are the specific differences between citizenship and human rights? Citizenship is essentially secular in origin and character. Originally a member of a city, it is now tied to sovereign states without reference to religion. We have seen that human rights are religious in origin – indeed Christian in origin through natural law theory, religious motivation behind anti-slavery [38]. While social citizenship is funded by taxation, there is no obvious taxation of recipients of human rights. There is much discussion of active citizenship and acts of citizens [20]. How are citizens empowered and how do they express that empowerment? Occupy Wall St, the Arab Spring, and the Umbrella Revolution would be examples. While citizens are active, human rights recipients are typically the passive subjects of violence. Of course, human rights recipients may struggle and protest for example against barriers to their migration from sovereign states. If human rights subjects have agency, why are they the recipients of human rights protection? Human rights subjects are typically victims not agents. One counter argument might be that victims of abuse have agency – they try to mobilize international support and intervention,they set up camps in safe areas; perhaps flight and escaping are agency.

The typical criticisms of human rights are well known: who pays for them? who enforces them? What are the duties? These problems are illustrated by the current refugee crisis in Europe. The first duty of a state is to provide for the security of its citizens and to guarantee their safe passage by issuing passports that are recognized by other states, but states are also bound by human rights obligations to respect the rights of refugees. We can understand the contradictions between these two systems of norms – security of citizens and the legitimate claims of asylum seekers by reference to different state responses to the general crisis of European states – migration, populism, and Brexit. The refugee crisis is of course not confined to Europe. Thousands of refugees, fleeing from gangsterism, drug abuse and political dictatorships in Honduras, Guatemala, and El Salvador, are being stopped at the Mexican-US border by thousands of American troops. With the endless flow of refugees arriving in small border towns along the Mexican–American border, the local authorities cannot cope with the demands on their fragile arrangements.

## Precarity and Vulnerability

In most western societies, social citizenship is no longer a comprehensive and effective safety net against unemployment, underemployment, poor health, old age, and unaffordable housing. It is rather the feckless individual of neo-liberal criticism who is now held responsible for his or her own plight rather than large-scale structural changes brought about by technological change, structural transformations of the economy with financialization or the negative consequences of outsourcing. Social citizenship was originally tied to Keynesian economics and (in the United Kingdom) to the National Health Service based on the Beveridge Report. These socio-economic assumptions have been swept aside by supply-side economics, the economic policies arising from Milton Friedman’s influence on economics at the University of Chicago (the so-called ‘Chicago Boys’), and by Thatcherism and Reaganomics. Critics of supply-side economics (a reduction on taxes over all) and trickle-down theory (a reduction in taxes of the rich) have argued that inequality has increased, and that the global fiscal crisis dramatically demonstrated the dangers of deregulation [9]. Although these changes have been taking place since the 1970s with the beginning of the neo-liberal agenda and the demise of economic policies based on Keynesian economic theories, it is the aftermath of the global financial crisis of 2007–8 that is critical to the contemporary conjuncture. Of course, this (often implicit or hidden) transformation of citizenship rights has also been matched by an erosion of duties. The Marshall version of citizenship was based on a system of contributory rights [44]. Military service (in some circumstances by conscription), payment of taxes, supporting a family (to produce the next generation of citizens), taking care of elderly parents, and involvement in the community (such as voluntary service) were duties that were attached to rights. In legal terminology, there was correlativity between rights and duties [47]. This framework has largely disappeared leaving only a vestige of the model of social citizenship. The modern citizen is more controversially defined as a denizen with a weak and uncertain relationship to civil society and the nation-state [50].

Marshall’s vision of citizenship in the 1950s has been radically transformed [25]).Indicators of these changes include the impact of multiculturalism, the political struggle for citizenship rights and the erosion of economic security through market-driven policy shifts [37], leading to the growth of enormous wealth and income inequalities that are reflected in the changing composition of elites [35].Changes in the nature of employment, the transformation of taxation, privatization of many basic services and the ageing of the population have undermined the traditional welfare state. Although tax reductions are popular with voters, lower taxes limit the capacity of governments to fund social services and maintain the infrastructure of a modern society. The cost of medical care has also increased with an ageing population, pharmaceutical costs, and the complexity of medical research. Modern medical education imposes large demands on the modern university and on the national budget [6]. One policy response to rising medical costs has been the growing privatization of health services. Another policy has been to erode or terminate pension rights [5].

The growing insecurity of many sectors of society has been captured by a new concept–the precariat [40]. The notion of ‘precarity’ attempts to capture the risks that are faced especially by young and underqualified citizens who find themselves unemployed or in positions with low wages and high employment insecurity. In the United Kingdom, the Institute for Fiscal Studies reported (5 October 2018) that such workers are the most at risk to the negative effects of economic and social change – or broadly speaking de-industrialization. Older men with low qualifications in process, plant and machinery work will be most exposed. Looking at women and men as a whole, whereas 10% of women workers will be in this risk category, for men it is 17%. After Brexit, the fall-out from the decline in the automotive manufacturing industry in the United Kingdom supports these assumptions.

One criticism of the Marshall approach was directed at its assumptions about gender. In fact, Marshall’s account served to define the working man of the post-war democracies. The male citizen could look forward, at least in principle, to continuous employment, a welfare system, a pension, and an income sufficient (with prudence and abstemiousness) to rent or buy a modest property and support a family with a wife and two children. The assumption behind Marshall’s vision of British society was that the wife typically stayed at home to care for her children and support her working husband. The whole system gave the man a clear and emphatic status and identity within the community. Work and hard-earned wages defined citizenship. In this sense, married women, whose domestic labour was unpaid and had no independent income, existed on the margins of citizenship.

Clearly this entire edifice has been undermined by economic change, the transformation of work, the erosion of traditional family life, the decline in fertility, by the changing balance of power in gender relationships, the globalization of the economy and by migration (often as one policy response to the decline in fertility). In most societies, conscription has been replaced by a combination of professional armies and private security companies. Consequently, men in precarious and insecure work face increasing difficulty finding a female partner to form a stable relationship leading to marriage, reproduction, and family formation. The commercial norms of modern romance, as celebrated in popular culture, have become increasingly expensive and demanding, requiring a fast motor car, opportunities for dining out, holidays in exotic places, fashionable clothing, and clubbing [19]. Although widely accepted as part of the modern utopia of romance, these norms place romantic dating beyond the income of males at the bottom of the employment ladder. The resentment we see in populism and aggressive masculinity–encouraged by a new breed of authoritarian political figures such as Trump, Putin, and Duterte–is a key consequence of the decline of the social and economic role of the traditional male worker and increasing expulsion of redundant middle class white-collar workers from secure fulltime work. The causal argument behind this general view of social change is that social and economic developments from the late 1970s have produced weak citizenship and growing precarity. Economic change has eroded the status of the fully-employed, heterosexual, working man who feels challenged by feminism, gay and lesbian politics, the elite, the apparent indifference of politicians to their constituents (the Washington swamp), the pink-collar economy, globalization, and attendant inflows of migrants. The disappearance of this masculine world produces insecurity, resentment and incivility, and these feed into the populist politics of the left behind or the precariat [12]. Time and place for different societies will give rise to variations on these themes. It is recognized that the impact of these changes varies by society. However, the idea of precarity gives a social perspective on the fundamental vulnerability of people even in the advanced world of western democracy. Once citizenship rights have been stripped of their social foundations, what is left behind? Can citizens appeal successfully to their rights claims against economic insecurity?

## Conclusion

My original interest in vulnerability was inspired by philosophical anthropology. This development took place in Germany in the late 1920s and continued until after World War II with philosophers such as Gehlen. The original inspiration came from Max Scheler who believed that philosophical research had to take on board the developments and findings of anthropology, history, biology, and sociology. His principal publication was *Die Stellung der Menschen in Kosmos* in 1927 and eventually translated in 1958 as *Man’s Place in Nature.* He was driven by a puzzle. Given all these scientific developments, the irony was that we don’t really know what ‘Man’ is. Arnold Gehlen’s *Der Mensch, seine Natur und Stellung in der Welt* in 1940 was directly inspired by Scheler and translated as *Man. His Nature and Place in the World* [15]. Gehlen’s basic view was that humans are by their very nature vulnerable, and they can only survive by collective arrangements based on institutions including legal provisions such as rights. We can summarize Gehlen’s philosophy in one sentence which is in turn the basis for my basic understanding of vulnerability: ‘Simply staying alive is man’s ultimate challenge’.
